# Comparative transcriptome analysis of the newly discovered insect vector of the pine wood nematode in China, revealing putative genes related to host plant adaptation

**DOI:** 10.1186/s12864-021-07498-1

**Published:** 2021-03-16

**Authors:** Zehai Hou, Fengming Shi, Sixun Ge, Jing Tao, Lili Ren, Hao Wu, Shixiang Zong

**Affiliations:** 1 Key Laboratory of Beijing for the Control of Forest Pests, Beijing Forestry University, Beijing, China; 2 Liaoning Provincial Key Laboratory of Dangerous Forest Pest Management and Control, Shenyang, China

**Keywords:** Cerambycidae, *Monochamus saltuarius*, Host adaptation, Transcriptional variation, *Pinus koraiensis*, *Pinus tabuliformis*

## Abstract

**Background:**

In many insect species, the larvae/nymphs are unable to disperse far from the oviposition site selected by adults. The Sakhalin pine sawyer *Monochamus saltuarius* (Gebler) is the newly discovered insect vector of the pine wood nematode (*Bursaphelenchus xylophilus*) in China. Adult *M. saltuarius* prefers to oviposit on the host plant *Pinus koraiensis*, rather than *P. tabuliformis*. However, the genetic basis of adaptation of the larvae of *M. saltuarius* with weaken dispersal ability to host environments selected by the adult is not well understood.

**Results:**

In this study, the free amino and fatty acid composition and content of the host plants of *M. saltuarius* larvae, i.e., *P. koraiensis* and *P. tabuliformis* were investigated*.* Compared with *P. koraiensis*, *P. tabuliformis* had a substantially higher content of various free amino acids, while the opposite trend was detected for fatty acid content. The transcriptional profiles of larval populations feeding on *P. koraiensis* and *P. tabuliformis* were compared using PacBio Sequel II sequencing combined with Illumina sequencing. The results showed that genes relating to digestion, fatty acid synthesis, detoxification, oxidation-reduction, and stress response, as well as nutrients and energy sensing ability, were differentially expressed, possibly reflecting adaptive changes of *M. saltuarius* in response to different host diets. Additionally, genes coding for cuticle structure were differentially expressed, indicating that cuticle may be a potential target for plant defense. Differential regulation of genes related to the antibacterial and immune response were also observed, suggesting that larvae of *M. saltuarius* may have evolved adaptations to cope with bacterial challenges in their host environments.

**Conclusions:**

The present study provides comprehensive transcriptome resource of *M. saltuarius* relating to host plant adaptation. Results from this study help to illustrate the fundamental relationship between transcriptional plasticity and adaptation mechanisms of insect herbivores to host plants.

**Supplementary Information:**

The online version contains supplementary material available at 10.1186/s12864-021-07498-1.

## Background

For insect herbivores, adaptation to host plants is crucial to their ability to colonize a variety of environments [1]. Host plants produce a variety of allelochemicals including various defense compounds that protect them against herbivores; meanwhile, insect herbivores have developed different means to struggle with the chemical barriers that deter them from feeding [2]. Owing to the variety of plant defense compounds, a generalist herbivorous insect has to overcome a range of chemical challenges [3]. The capacity of herbivores to metabolize and detoxify plant chemicals is considered as one of their main evolutionary adaptations [4]. Although the importance of insect adaptation to plant chemicals is widely recognized, the underlying genetic mechanisms in response to their host plant defenses are still insufficient [3, 4].

The pine wood nematode (PWN; *Bursaphelenchus xylophilus*) is a plant parasitic nematode and major cause of pine wilt disease in Asia and Europe [5]. The transfer of PWN between host trees is mediated by insect vectors, e.g., various species of *Monochamus* beetles [6, 7]. In Asia, PWN infection mainly occurs during feeding and oviposition of the Japanese pine sawyer *Monochamus alternatus* Hope [5]. Besides *M. alternatus*, the Sakhalin pine sawyer *M. saltuarius* (Gebler) (Coleoptera: Cerambycidae) is another important insect vector of PWN in Japan [8] and Korea [9]. Recently, *M. saltuarius* has also been confirmed as an effective vector of PWN in Liaoning Province, China [10–12]. The Korean white pine *Pinus koraiensis* Siebold & Zucc, a tree species of economic importance [13], was found to be a natural host for the PWN in the Republic of Korea in 2006, and *M. saltuarius* transmitted PWN to *P. koraiensis* [14]. Similarly, *M. saltuarius* was found to transmit PWN to *P. koraiensis* in China [10, 12]. In addition, Han et al. [15] investigated the feeding and oviposition preference of *M. saltuarius* among eight tree species, including *P. koraiensis*, and they found that the highest feeding amount and oviposition preference were related to *P. koraiensis*. Similarly, Pan et al. [16] reported that adults of *M. saltuarius* preferred *P. koraiensis* than *P. tabuliformis* Carr. and *Larix kaempferi* (Lamb.) Carr. based on feeding behavior. Volatiles produced by host plants, e.g., α-pinene, are known to attract *Monochamus* spp. [17, 18]. Adults of *M. saltuarius* can be attracted by terpenes emitted from the host plant *P. koraiensis* for feeding and oviposition [19, 20]. In addition, host volatiles also play an important role in the mating location of longhorned beetles [21]. Therefore, the distribution pattern in the adults of *M. saltuarius* can be affected by host volatiles.

In many organisms, including insect species, larvae/nymphs are unable to disperse far from the oviposition site selected by the mother [22]. Consequently, oviposition host selection can strongly impact both the survival and the spatial distribution of a species [23], and the structure and composition of animal communities [24]. Female adults of *M. saltuarius* lay their eggs on the bark of pine trees. After hatching, the larvae feed on the inner cambium bark and outer sapwood. Because adults of *M. saltuarius* prefer *P. koraiensis* over *P. tabuliformis* for feeding and oviposition, coupled with the weakened dispersal ability at the instar stage, the larvae of *M. saltuarius* may be confronted with different chemical challenges posed by their different hosts. However, the molecular mechanisms underlying host plant adaptation of *M. saltuarius* larvae are largely unknown.

Detecting transcriptional changes related to host adaptation is a vital link to understand plant-insect interactions [3, 25, 26]. Previous studies have proved that transcriptional plasticity of insects was related to diet. For instance, research on host adaptation in cactophilic flies, e.g., *Drosophila mojavensis*, *D. buzzatii,* and *D. mettleri*, have identified a series of genes associated with carbohydrate metabolism, cellular energy production, xenobiotic metabolism, and stress response [2, 25, 27]. Research on the striped stem borer *Chilo suppressalis*, Zhong et al. [26] identified several genes involved in host plant adaptation processes, including digestion and detoxification. Larvae of the Asian long-horned beetle *Anoplophora glabripennis* modulate a subset of genes associated with digestion when fed on a nutrient-poor, compared to a nutritious diet [28]. In addition, Scully et al. [29] showed that feeding on two appropriate host plants (*Acer* spp. and *Populus nigra*) modified the expression levels of multicopy genes involved in digestion and detoxification in *A. glabripennis*. Recently, Hou & Wei [30] examined the transcriptional changes of the cicada *Subpsaltria yangi*, on a varied diet of different host plants. The authors suggested that gene expression changes, relating to digestion, detoxification, oxidoreductase metabolism, and stress response, may be a vital adaptation to diet and habitat.

With the rapid development of sequencing technology, research into the insect transcriptome is increasing [31, 32]. However, de novo transcriptome assembly represents a challenge for non-model insect species, because it generally relies on the use of short cDNA sequences (such as Illumina technology). Recently, single-molecule real-time sequencing (SMRT-seq) technology has been applied to generate long sequence reads, allowing the production of full-length transcripts without assembly algorithms [33]. SMRT-seq has been reported to provide inaccurate information on genes, which could be calibrated based on Illumina reads from matched samples [34]. Therefore, the combination of SMRT-seq and Illumina RNA-seq can be used to obtain comprehensive genetic information, including for the detection of gene isoforms and functional variants [35, 36].

In the present study, the free amino and fatty acid composition and content of the two host plants of *M. saltuarius*, categorized as either the “preferred” *P. koraiensis* or “non-preferred” *P. tabuliformis*, was investigated. The genome-wide transcriptional profiles of *M. saltuarius* larvae feeding on *P. koraiensis* and *P. tabuliformis* was compared by combining SMRT-seq and Illumina RNA-seq analysis. Our aim was to identify differentially expressed genes (DEGs) in *M. saltuarius* relating to host plant adaptation based on diet. The results provide new information for further research on the mechanisms underlying transcriptional plasticity and adaptation of insect herbivores to different host plants. Furthermore, understanding the molecular differences of *M. saltuarius* when feeding on different hosts may provide significant enlightenment for the arrangement of host resistance in the control of PWN transmission.

## Results

### Host plant free amino and fatty acid composition and content

**Fig. 1:**
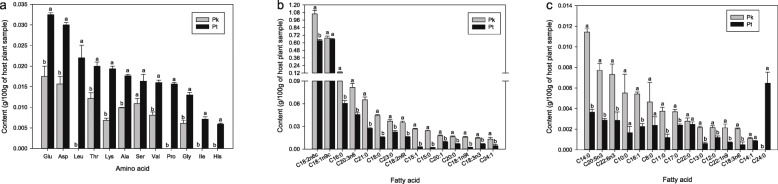
Amino acid and fatty acid composition and content between host plants *Pinus koraiensis* and *P. tabuliformis*. **a** Amino acid. **b**, **c** Fatty acid. Glu, glutamic acid; Asp, aspartic acid; Leu, leucine; Thr, threonine; Lys, lysine; Ala, alanine; Ser, serine; Val, valine; Pro, proline; Gly, glycine; Ile, isoleucine; His, histidine. C18:2n6c, linoleic acid; C18:1n9c, oleic acid; C16:0, palmitic acid; C20:3n6, Dihomo-γ-linolenic acid; C21:0, Heneicosylic acid; C18:0, Stearic acid; C23:0, Tricosanoic acid; C18:2n6t, Linoelaidic acid; C15:1, 10c-pentadecenoic acid; C15:0, Pentadecanoic acid; C20:1, Eicosenoic acid; C20:0; Arachidic acid; C18:1n9t, Elaidic acid; C18:3n3, α-Linolenic acid; C24:1, Nervonic acid; C14:0, Myristic acid; C20:5n3, Eicosapentaenoic acid (EPA); C22:6n3, Docosahexaenoic acid (DHA); C10:0, Decanoic acid; C16:1, Palmitoleic acid; C8:0, Octanoic acid; C11:0, Undecanoic acid; C17:0, Margaric acid; C22:0, Behenic acid; C13:0, Tridecylic acid; C12:0, Lauric acid; C22:1n9, Erucic acid; C18:3n6, γ-Linolenic acid; C14:1, Myristoleic acid; C24:0, Lignoceric acid. Data are shown as mean ± SE. Different letters represent significant statistical difference at the 0.05-level

Eight free amino acids were found in *P. koraiensis*, including glutamic acid (Glu), aspartic acid (Asp), threonine (Thr), lysine (Lys), alanine (Ala), serine (Ser), valine (Val), and glycine (Gly). Twelve free amino acids were found in *P. tabuliformis*, i.e., Glu, Asp, leucine (Leu), Thr, Lys, Ala, Ser, Val, proline (Pro), Gly, isoleucine (Ile), and histidine (His). The main free amino acids in the two host plants were Glu and Asp. Compared with *P. koraiensis*, *P. tabuliformis* had a substantially higher content of most free amino acids (Fig. 1a).

Twenty-nine and thirty fatty acids were detected in *P. koraiensis* and *P. tabuliformis*, respectively. The predominant fatty acids present in the two host plants were linoleic (C18:2n6c), oleic (C18:1n9c), and palmitic acids (C16:0). Compared with *P. tabuliformis*, *P. koraiensis* had a substantially higher content of most fatty acids (Fig. 1b, c).

### Combined sequencing of *Monochamus saltuarius* transcripts

The full-length transcriptome of *M. saltuarius* was produced based on the pooled RNA from the six samples of *M. saltuarius* using the PacBio Sequel II platform. A total of 22.36 Gb subreads was produced by one SMRT cell from the PacBio library (Table 1). The subreads from the same polymerase read sequence formed a circular consensus sequence (CCS), which yielded 284,546 CCSs with an average read length of 2583 bp, and the length distribution of the CCS reads is shown in Additional file 1: Figure S1a. Among them, 234,939 full-length non-chimera (FLNC) reads (82.57% of CCSs) were obtained, and the length distribution of the FLNC reads is shown in Additional file 1: Figure S1b. In total, 48,361 consensus isoforms with a mean length of 3122 bp were detected through the Iterative Clustering for Error Correction (ICE), including 46,082 polished high-quality isoforms (Table 1). The 48,361 consensus isoforms were corrected based on the Illumina RNA-seq data (Table 2) to improve quality. After removing redundant sequences and a cluster of low-quality transcripts using CD-HIT (c = 0.99), a total of 32,304 non-redundant transcripts with a mean length of 3290 bp were obtained, which were further annotated for downstream analysis. The completeness of our transcript dataset was assessed with benchmarking universal single-copy orthologs (BUSCO), and the result revealed that this dataset consisted of 89.5% complete and 1.9% partial BUSCO orthologs (Additional file 2: Figure S2).

**Table 1:** Summary for the full-length transcriptome of *Monochamus saltuarius* analyzed with the PacBio Sequel II platform

Library	1–6 kb
SMRT cell	1
Subreads base (G)	22.36
Number of CCS	284,546
Read bases of CCS	735,095,084
Mean read length of CCS	2583
Mean number of passes	36
Number of undesired primer reads	37,473
Number of filtered short reads	21
Number of full-length non-chimeric reads	234,939
Number of consensus isoforms	48,361
Average consensus isoforms read length (bp)	3122
Number of polished high-quality isoforms	46,082
Number of polished low-quality isoforms	1917
Number of non-redundant transcripts	32,304

**Table 2:** Illumina-sequencing data analysis results

ID	Read number	Base number	GC content (%)	Q30 (%)	Uniquely mapped reads (%)	Reads mapped to multiple loci (%)	Reads mapped to many loci (%)
Pk1	19,908,903	5,943,030,832	41.98	93.18	36.57	42.86	6.13
Pk2	20,814,420	6,198,988,604	42.01	93.49	35.25	44.10	7.28
Pk3	19,898,375	5,936,828,046	42.02	93.59	35.06	43.17	7.80
Pt1	20,331,901	6,064,964,864	42.17	93.38	37.87	42.08	6.29
Pt2	21,214,464	6,338,924,668	42.65	93.29	35.45	43.17	8.99
Pt3	21,507,511	6,426,164,508	42.49	93.30	38.79	41.75	6.28

Q30: proportion of nucleotides with quality value larger than 30 in reads. This means that the base call accuracy (i.e., the probability of a correct base call) is 99.9%

For Illumina sequencing, 36.91 Gb high quality sequences were obtained from the six mRNA samples of *M. saltuarius*. The guanine-cytosine (GC) content of data sequenced from the six libraries was ~ 42%, and the percentage of reads with an average quality score > 30 was above 93% (Table 2). This result indicated that the accuracy and quality of the sequenced data were sufficient for further analysis. The Illumina sequencing reads were not assembled alone because more than 85% of them mapped to the 32,304 non-redundant transcripts (Table 2).

### Functional annotation

To obtain a comprehensive functional annotation of the full-length transcriptome of *M. saltuarius*, a total of 32,304 non-redundant transcripts were aligned with different databases (Table 3). A total of 29,798 transcripts (92.24%) were annotated in at least one database. The transcripts were mostly annotated by the Nr (NCBI non-redundant protein sequences) database (29,113; 90.12%) (Additional file 3: Table S1). The highest percentage of unigene sequences were matched with *Anoplophora glabripennis* (83.18%), followed by *Leptinotarsa decemlineata* (2.04%), *Tribolium castaneum* (1.80%), and *Callosobruchus maculatus* (1.51%) (Additional file 4: Figure S3).

**Table 3:** Non-redundant transcripts identified from different databases

Annotated databases	Number
Nr	29,113
KEGG	27,077
eggNOG	26,783
NT	25,234
Pfam	22,435
KOG	20,446
Swiss-Prot	20,412
GO	13,144
At least one database	29,798
All database	9262

In total, 13,144 transcripts were assigned Gene Ontology (GO) terms, which were classified into the three major GO categories (Additional file 5: Figure S4). For the biological process classification, genes involved in ‘cellular process’, ‘single-organism process’, and ‘metabolic process’ were highly represented. For the cellular component, the major categories were ‘cell’, ‘cell part’, and ‘organelle’. For the molecular function classification, ‘binding’ was the most enriched GO term, followed by ‘catalytic activity’. Kyoto Encyclopedia of Genes and Genomes (KEGG) analysis shows that the matched 27,077 transcripts are assigned into 336 pathways. The most well-represented metabolic pathways are involved in ‘global and overview maps’, ‘carbohydrate metabolism’, ‘lipid metabolism’, and ‘amino acid metabolism’ (Additional file 6: Figure S5).

### Transcription factor identification, and lncRNA and SSR prediction

**Fig. 2:**
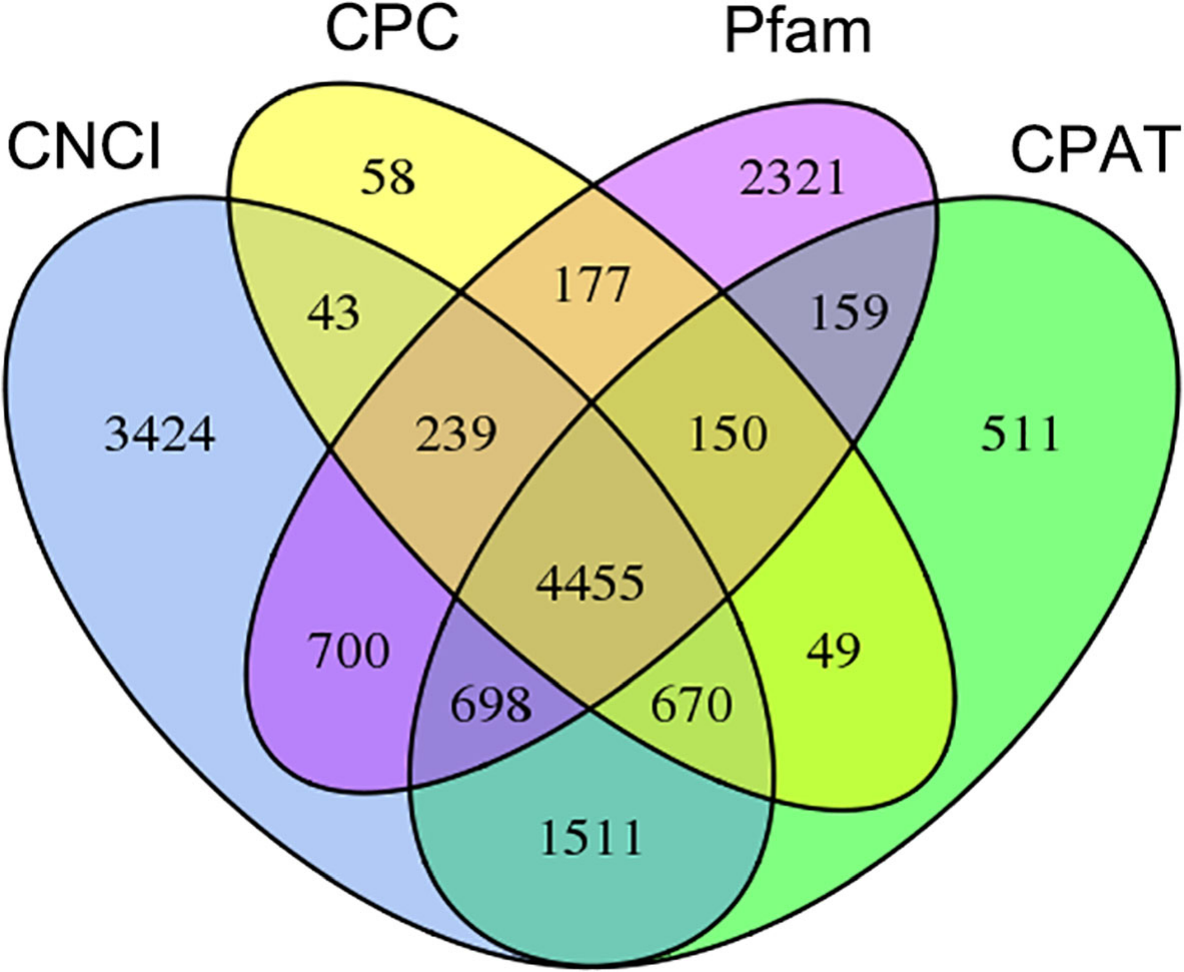
Venn diagram of the number of lncRNAs predicted using coding-non-coding index (CNCI), coding potential calculator (CPC), coding potential assessment tool (CPAT) and protein family (Pfam) database

A total of 1833 transcription factors (TFs) were identified, with zf-C2H2 accounting for the largest proportion of the known TF families, followed by ZBTB (Additional file 7: Figure S6). Four coding potential analysis methods were used to predict the long non-coding RNA (lncRNA), including coding potential calculator (CPC), coding-non-coding index (CNCI), coding potential assessment tool (CPAT), and protein family (Pfam) database. The numbers of lncRNAs predicted from non-redundant transcripts by CPC, CNCI, CPAT, and Pfam were 5841, 11,740, 8203 and 8899, respectively (Fig. 2). The intersection of these four results yielded 4455 lncRNA transcripts (Fig. 2). The average length of the lncRNA transcripts was 2863 bp.

In this study, 31,530 transcripts were scanned by MISA (MIcroSAtellite identification tool). A total of 17,164 simple sequence repeats (SSRs) were identified from 10,929 transcripts, including six major subtypes: mononucleotide (12,875), di-nucleotide (1838), tri-nucleotide (2231), tetranucleotide (187), penta-nucleotide (21), and hexa-nucleotide (12). Among them, 1398 SSRs were present in the compound formation (Additional file 8: Table S2).

### DEG analysis

We evaluated the differences in gene expression between the population feeding on *P. koraiensis* and *P. tabuliformis*. It resulted in 2166 DEGs identified in the larvae of *M. saltuarius* feeding on *P. tabuliformis* (Pt) compared with *P. koraiensis* (Pk), including 970 upregulated genes and 1196 downregulated genes (Additional file 9: Table S3; Additional file 10: Figure S7).

**Fig. 3:**
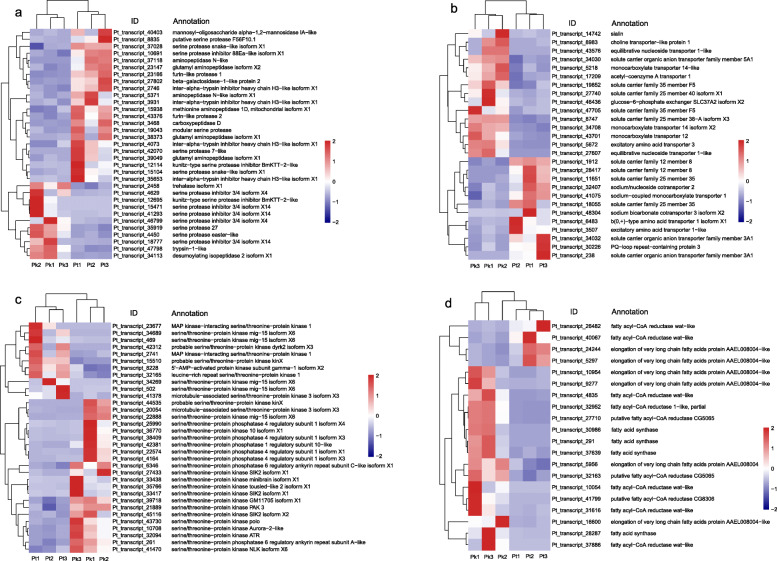
Heatmap of normalized FPKM of DEGs related to **a** digestion, **b** putative osmoregulation, **c** sensing availability of nutrients and energy, **d** fatty acid and lipid metabolism. The Z-score represents the deviation from the mean by standard deviation units. The firebrick color indicates upregulated expression, whereas the navy color indicates downregulated expression. FPKM: fragments per kilobase of transcript per million fragments mapped; Pk: the larvae feeding on *Pinus koraiensis*; Pt: the larvae feeding on *P. tabuliformis*

In this study, transcriptional changes related to host plant adaptation in *M. saltuarius* was the main focus. We identified 21 DEGs associated with digestion in the comparative set ‘Pt vs Pk’, encoding three carbohydrases and 18 proteases. Most of these were upregulated in *P. tabuliformis* (Fig. 3a). In addition, we identified 12 DEGs related to protease inhibitor, including eight serine proteases and four trypsin inhibitors (Fig. 3a).

Solute carriers (SLC) are a group of membrane transport proteins, which mediate the transport of various substrates across cells, including ions, nucleotides, sugars, and amino acids. We identified 27 DEGs encoding solute carriers in the comparative set ‘Pt vs Pk’ (Fig. 3b), which may mediate the influx or efflux of substance and involve in the osmoregulation in the host adaptation of *M. saltuarius*.

The serine/threonine protein kinase (STK) target of rapamycin, a central element of an evolutionarily conserved eukaryotic signaling pathway, is known to act as a central regulator of cell metabolism and to respond to growth factors and nutritional status. In the present study, we identified 25 DEGs encoding STKs and seven DEGs encoding serine/threonine phosphatases (STPs) (Fig. 3c). In addition, AMP-activated protein kinase (AMPK) serves as an important regulator of cellular metabolism and energy balance. One gene encoding AMPK was found upregulated in the comparative set ‘Pt vs Pk’ (Fig. 3c).

Fatty acids are a significant energy store for insects. Four DEGs encoding fatty acid synthase (FAS) were identified in the comparative set ‘Pt vs Pk’ (Fig. 3d). In addition, six genes encoding elongation of very long chain fatty acids protein (ELOVL) were differentially expressed in the population feeding on *P. tabuliformis* when compared with *P. koraiensis* (Fig. 3d). Besides FAS and ELOVL, fatty acyl-CoA reductase (FAR), which can convert fatty acids to alcohols, performs a crucial role in lipid synthesis and metabolism. Ten DEGs encoding FARs were identified in the comparative set ‘Pt vs Pk’, including eight upregulated in the population feeding on *P. tabuliformis* (Fig. 3d).

**Fig. 4:**
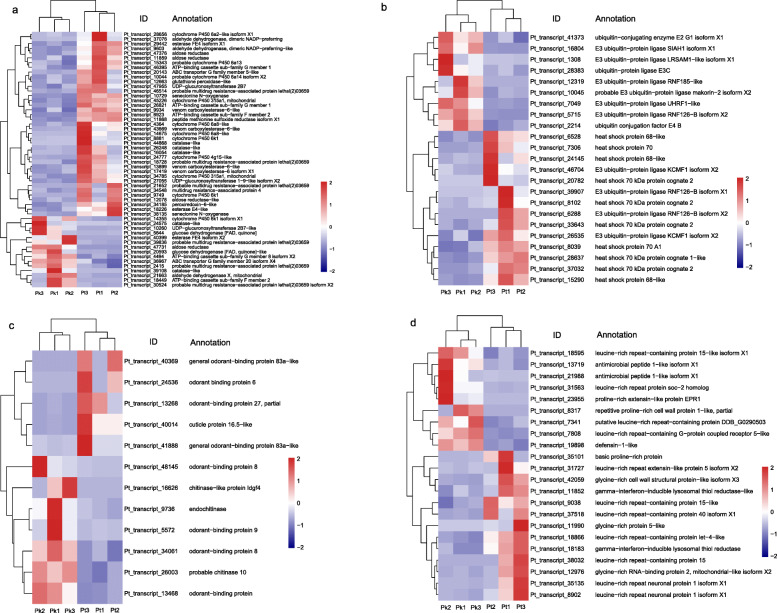
Heatmap of normalized FPKM of DEGs related to **a** detoxification and oxidation-reduction, **b** stress response, **c** structural and general odorant binding proteins, **d** antibacterial and immune response. The Z-score represents the deviation from the mean by standard deviation units. The firebrick color indicates upregulated expression, whereas the navy color indicates downregulated expression. FPKM: fragments per kilobase of transcript per million fragments mapped; Pk: the larvae feeding on *Pinus koraiensis*; Pt: the larvae feeding on *P. tabuliformis*

Insect herbivores should be able to deal with defense compounds and adverse environment when obtaining nutrients from their host plants. In the present study, detoxification-related DEGs were identified, including 11 cytochrome P450 monooxygenases (P450s), three UDP-glycosyltransferases (UGTs), seven carboxylesterases (CEs), and 14 ATP-binding cassette (ABC) transporters (Fig. 4a). Among which, ten P450s, two UGTs, six CEs, and eight ABC transporters were upregulated in the population feeding on *P. tabuliformis* compared with *P. koraiensis* (Fig. 4a). We identified three aldehyde dehydrogenases (ALDHs), four aldose reductases, two senecionine N-oxygenases (SNOs), and two glucose dehydrogenases, most of which were upregulated in the population feeding on *P. tabuliformis* (Fig. 4a)*.* We also found that DEGs encoding peroxidase, i.e., five catalases (CAT), one glutathione peroxidase (GPx)-like, and one peroxiredoxin (Prx)-6-like, were mainly upregulated in the population feeding on *P. tabuliformis* compared with *P. koraiensis* (Fig. 4a). These genes might involve in defense response against oxidative stress, e.g., reactive oxygen species (ROS) intake in the feeding behavior. In addition, we found that one *peptide methionine sulfoxide reductase* (*MSRA*) gene was upregulated in the population feeding on *P. tabuliformis* (Fig. 4a), which may help repair proteins inactivated by oxidation.

Heat shock family 20 and 70 proteins serve as chaperones for damaged proteins in wood-consuming insects. In the present study, ten genes encoding heat shock proteins (Hsp), including seven Hsp70 and three Hsp68, were upregulated in the population feeding on *P. tabuliformis* compared with *P. koraiensis* (Fig. 4b). Additionally, other DEGs involved in the stress response were also identified, including 11 genes encoding E3 ubiquitin ligase, one gene encoding ubiquitin conjugating enzyme E2G1, and one gene encoding ubiquitin conjugation factor E4B (Fig. 4b).

Plant-derived compounds may interfere with the production of chitin and cuticular protein, which compels insect herbivores to adjust the production of these structural constituents. In the present study, three genes encoding chitinase, and one gene encoding cuticular protein were differentially expressed in the population feeding on *P. tabuliformis* compared with *P. koraiensis* (Fig. 4c). Additionally, eight genes encoding odorant-binding protein/general odorant-binding protein (OBP/GOBP) were differentially expressed in the comparative set ‘Pt vs Pk’ (Fig. 4c).

Insects always interact with a wide array of pathogens, e.g., pathogenic bacteria. Insects can prevent infection by synthesizing antibacterial proteins such as cecropin, insect defensin, large glycine-rich protein, small proline-rich protein, and lysozyme [37, 38]. In addition, leucine-rich repeat (LRR)-containing proteins play important roles in pathogen-associated molecular pattern recognition to fight infection by pathogens. In the present study, we found two genes encoding antimicrobial peptide 1-like isoform X1, one gene encoding defensin-1-like, three genes encoding glycine-rich proteins, three genes encoding proline-rich proteins, and two genes encoding gamma-interferon-inducible lysosomal thiol reductase (GILT), were differentially expressed in the comparative set ‘Pt vs Pk’ (Fig. 4d). Additionally, we identified 11 DEGs encoding LRR containing proteins in the population feeding on *P. tabuliformis* compared with *P. koraiensis* (Fig. 4d).

### Enrichment pathway analysis of DEGs

**Fig. 5:**
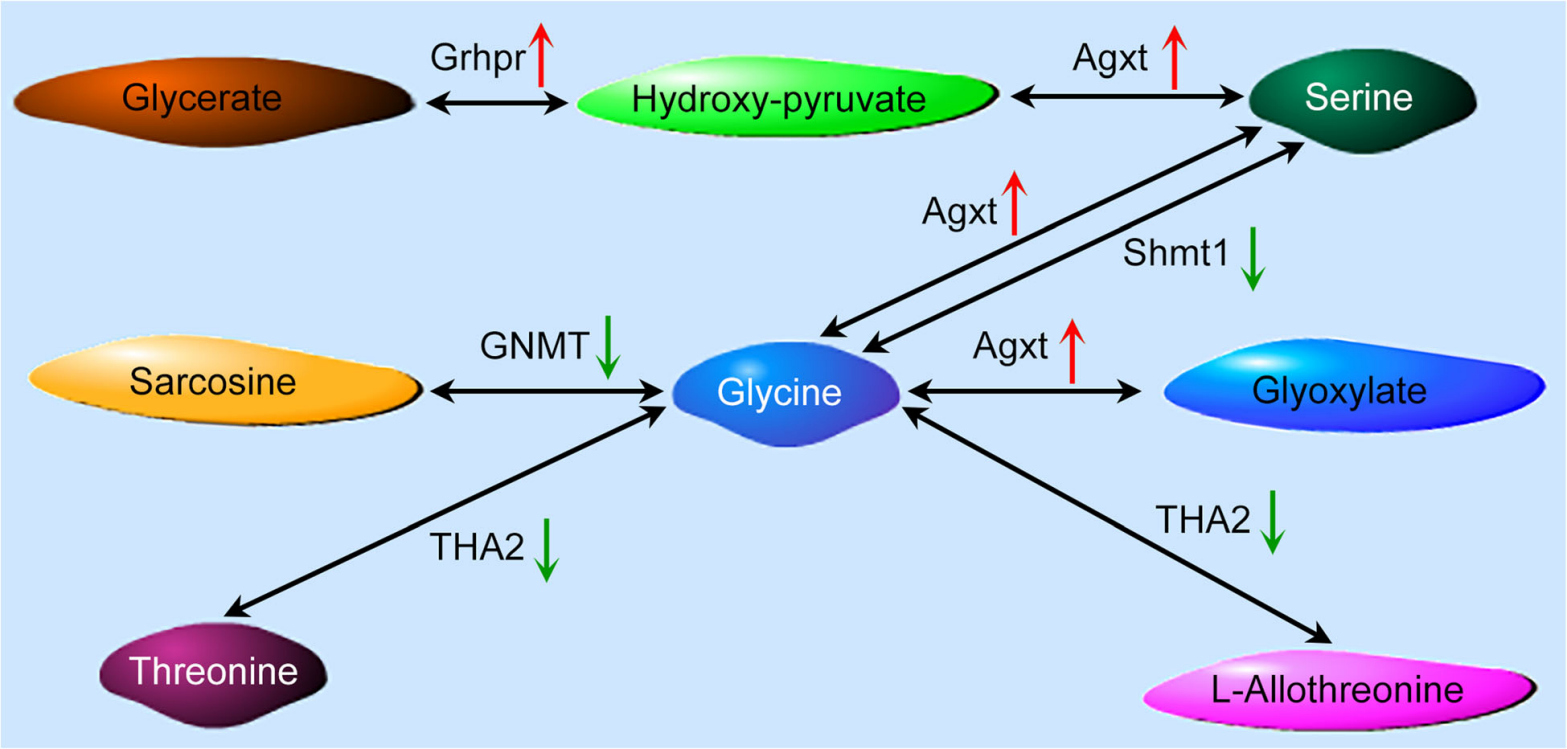
DEGs involved in glycine, serine, and threonine metabolic pathways. Red and green arrows indicate significantly up- and downregulated expression, respectively

To analyze functions of the DEGs, all were mapped to terms in the KEGG database. KEGG pathways with a *P*-value < 0.05 are provided in Additional file 11: Table S4, including insect hormone biosynthesis (ko00981), tryptophan metabolism (ko00380), cysteine and methionine metabolism (ko00270), and glycine, serine, and threonine metabolism (ko00260) (Fig. 5). As mentioned above, the content of glycine and threonine in *P. tabuliformis* was higher than that in *P. koraiensis* (Fig. 1a). This suggests that the “glycine, serine and threonine metabolism” pathway may play a key role in the host plant adaptation of *M. saltuarius*.

Following this, GO term enrichment analysis was performed. The strongest changes in the top 20 GO categories are shown in Additional file 12: Figure S8, including “oxidoreductase activity (GO:0016491)” of molecular function, and “alpha-amino acid metabolic process (GO:1901605)” of biological process.

### Validation of RNA-seq data by qRT-PCR

**Fig. 6:**
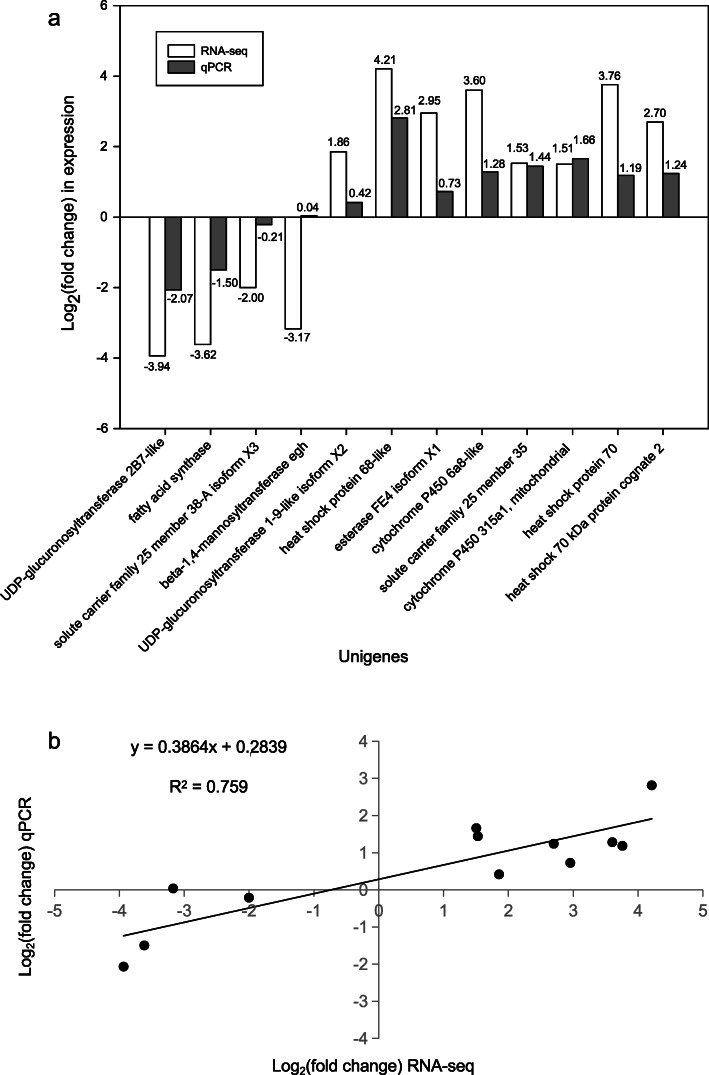
Validation of sequencing data by qRT-PCR. **a** The expression changes for each candidate gene as measured by RNA-seq and qRT-PCR. **b** Scatter plot showing correlation between log_2_(fold change) obtained via RNA-seq and qRT-PCR. Log_2_(fold change) qPCR was calculated by −∆∆Ct = − [(Ct_Pt_ – Ct_nom_) – (Ct_Pk_ – Ct_nom_)]

To validate the RNA-seq results, the relative expression levels of 12 selected genes were analyzed by real-time quantitative PCR (qRT-PCR). The genes and primers used for qRT-PCR are shown in Additional file 13: Table S5. Among the 12 genes, the majority showed a consistent expression pattern between RNA-seq and qRT-PCR (Fig. 6a). The correlation analysis results for these detected DEGs are as follows: y = 0.3864x + 0.2839, and R^2^ = 0.759 (Fig. 6b), indicating that RNA-seq data were reliable.

## Discussion

Herbivorous insects are hypothesized to express distinctive digestive enzymes to feed on host plants with different nutritional values [39, 40]. Indeed, some metabolism-related genes are differentially expressed to adapt to the different biochemical compositions of host plant diets [2, 25, 41]. For instance, Chikate et al. [42] revealed diet-specific protease expression patterns in the cotton bollworm *Helicoverpa armigera* responding to nutritionally distinct host plants. They suggested that serine proteases play a crucial role in this polyphagous insect to adapt to a diet of many different host plants [42]. The butterfly larvae of *Polygonia c-album* were demonstrated to adapt similarly to host plant diet [43]. In order to examine genes related to host plant utilization, Mason et al. [28] compared gene expression changes of the larvae of *Anoplophora glabripennis* feeding on a preferred host (the sugar maple) to those consuming a nutrient-rich artificial diet. Recently, Scully et al. [29] examined how feeding on two susceptible (*Acer* spp. and *Populus nigra*) and a resistant host (*Populus tomentosa*) affected the gene expression of *A. glabripennis*.

In our present study, the two host plants differed in their free amino and fatty acid composition and content; thus, they were expected to have different nutritional values for *M. saltuarius*. In particular, the glycine and threonine content in the host plant *P. koraiensis* was higher than that in *P. tabuliformis*. KEGG pathway analysis of DEGs identified several pathways associated with amino acid metabolism, including glycine, serine and threonine metabolism. This result suggests that the different nutritional quality between the two hosts may require *M. saltuarius* to express different digestive enzymes.

The serine/threonine kinase TOR (target of rapamycin) serves as a central regulator of cell growth and cellular energy [44, 45]. Both protein kinases and their cognate phosphatases participate in sensing external stimuli [46–48]. In addition, AMPK is also known as the “energy sensor” of a cell [49]. Interestingly, AMPK and TOR are functionally interconnected, opposing signaling pathways associated with sensing the status of nutrients and energy [50]. In the present study, the genes encoding AMPK, STKs, and STPs, may be relevant to host adaptation of *M. saltuarius* by playing an important role in sensing the availability of nutrients and energy for the regulation of cell growth.

Fatty acids are important energy stores in insects [51] with FAS being a key enzyme for its synthesis. Animals can gain fatty acids from their diet, then generate saturated and monounsaturated fatty acids through FAS-catalyzed synthesis [52–54]. Fatty acid synthesis includes cycles of four reactions, in which each cycle extends an initial acetyl-CoA by two carbons. The cycles can be repeated up to seven times to generate palmitic acid (C16:0) [55]; further growth requires elongases of long or very long chain fatty acids [56]. In addition, the FAR gene family, which can convert fatty acids to fatty alcohols, also performs a crucial role in lipid synthesis and metabolism. In insects, fatty alcohols can act as precursors in the production of pheromones and cuticular hydrocarbons [57, 58]. For instance, Li et al. [59] reported that FARs are requisite for cuticle shedding, and are involved in cuticular hydrocarbon production in the destructive rice pest *Nilaparvata lugens.* In the present study, a substantially higher content of most fatty acids was found in the host plant *P. koraiensis* compared with *P. tabuliformis.* Moreover, four, six, and ten genes encoding FAS, ELOVL, and FAR, respectively, were differentially expressed, suggesting that they may be involved in fatty acid metabolism when *M. saltuarius* is confronted with different fatty acid content between the two host plants.

Besides extracting nutrients from host plants, insects need to cope with toxic chemical deterrents produced by their hosts. For instance, *P. tabuliformis* can produce defensive monoterpene, in which α-pinene is the most abundant [60]. Besides α-pinene, volatile organic compounds (VOCs) from *P. tabuliformis* also includes limonene, β-pinene, and α-caryophyllene [61]. Xu et al. [62] found that the VOCs from *P. koraiensis* included α-pinene, β-pinene, and sabinene. Insects can successfully survive on their host plants by producing detoxifying enzymes, e.g., P450s, UGTs, and CEs [25, 30, 41]. In the present study, we found that 11 P450, seven CE, and three UGT, and most of them were upregulated in the larvae feeding on *P. tabuliformis* compared with *P. koraiensis*. Also involved in detoxification are ABC transporters, which act as membrane-bound proteins for the transport of various substrates across the lipid membrane, including drugs and insecticides [63, 64]. In our study, 14 genes encoding ABC transporters were differentially expressed, including the up-regulation of eight, in larvae feeding on *P. tabuliformis* compared with *P. koraiensis*, indicating that these genes may involve in detoxification metabolism during the host plant adaptation of *M. saltuarius*.

In our study, we found that three, two, and six genes encoding aldehyde dehydrogenases, senecionine N-oxygenases, and aldo-keto reductases (AKRs), respectively, were differentially expressed, suggesting their involvement in plant chemical detoxification by *M. saltuarius.* The aldehyde dehydrogenases are oxidizing enzymes that are involved in detoxification of both exogenous and endogenous aldehydes [65]. In addition, senecionine N-oxygenase, a flavin-dependent monooxygenase, may help insects cope with pyrrolizidine alkaloids, as observed in the larvae of the European cinnabar moth *Tyria jacobaeae* [66]. The AKRs are a superfamily of dehydrogenases/reductases that catalyze the synthesis and detoxification of carbonyls [67]. Previously, AKRs have been hypothesized to play an important role in the degradation of woody tissue [28]. In this study, we therefore speculate that genes encoding AKRs may not only be involved in detoxification but may also assist *M. saltuarius* to digest woody tissue.

Insect herbivores are frequently challenged with ROS which are a by-product from the metabolism of molecular oxygen [68]. Some herbivorous insects have evolved a protective response to ROS by producing detoxifying enzymes, including glutathione peroxidase (GPx), catalase (CAT), superoxide dismutase (SOD), and ascorbate peroxidase [69]. For instance, in a serious pest on wheat crops (the Hessian fly *Mayetiola destructor*), when feeding on resistant wheat seedlings, the larvae upregulates expression of phospholipid glutathione peroxidases, catalases, and superoxide dismutases, to counteract ROS [68]. In our study, we found that five genes encoding CATs, one *GPx-like*, and one *Prx6-like* gene, were upregulated in the larvae feeding on *P. tabuliformis* compared with *P. koraiensis*, suggesting that they may involve in the defense against ROS intake during feeding.

As well as battling toxic chemicals from host plants, insects also must deal with a wide array of pathogens. Insects combat infection by mounting a powerful immune response [70]. Insect immune system contains two major aspects: the cellular and humoral responses [71]. The humoral response includes synthesis of antimicrobial proteins [72], which are grouped into five main types: cecropins, insect defensins, large glycine-rich proteins, small proline-rich proteins, and lysozymes [38]. The LRR is a highly conserved motif usually consisting of 20–30 residues rich in leucine, and a LRR domain, which is an important binding component for immune-related proteins [73]. In the insect *Manduca sexta*, a soluble, extracellular leucine-rich repeat protein (leureptin) could bind to bacterial lipopolysaccharide (LPS) and involve in hemocyte responses to bacterial infection [74]. GILT is involved in the bacterial immune response in various organisms [75]. Recently, empirical studies demonstrated that pinewood nematode infection increased the microbial diversity in pines [76, 77]. In the present study, genes encoding antimicrobial peptide 1-like isoform X1, defensin-1-like, glycine-rich proteins, proline-rich proteins, LRR domain containing protein, and GILT, were differentially expressed by *M. saltuarius* larvae, coupled with the difference in host preference of adults acting as vector of PWN, suggesting that larvae of *M. saltuarius* might confront with different bacterial challenges in their host environments.

Gene expression patterns identified in this study, might not just be altered by differential host plant diets, but also genetic variations in the sample populations [78], and local environments (i.e., temperature) [79]. In the present study, the two sample sites were approximately 40 km apart; thus, they may share similarities in their natural environment. Molecular identification of cytochrome c oxidase subunit I (*COI*) sequence distances of sampled individuals from the two sites were between 0.0 and 0.00897, indicating little genetic divergence between *M. saltuarius* populations. Previously, similar results have been reported from comparable sampling strategies [26, 30, 80]. Therefore, the different gene expression patterns detected in the present study may be mainly caused by host plant diet. Further research is needed to verify that the DEGs detected in our analysis are due to host adaptation mechanisms in *M. saltuarius*.

## Conclusions

In this study, firstly, we investigated the free amino and fatty acid composition and content of the host plants of *M. saltuarius* larvae, i.e., *P. koraiensis* and *P. tabuliformis.* Compared with *P. koraiensis*, *P. tabuliformis* had a substantially higher content of various free amino acids, while the opposite trend was detected for fatty acid content. Then, we compared the transcriptional profiles of larval populations feeding on *P. koraiensis* and *P. tabuliformis* using PacBio Sequel II sequencing combined with Illumina sequencing. The results showed that genes relating to digestion, fatty acid synthesis, detoxification, oxidation-reduction, and stress response, as well as nutrients and energy sensing ability, were differentially expressed, possibly reflecting adaptive changes of *M. saltuarius* in response to different host diets. Additionally, genes coding for cuticle structure were differentially expressed, indicating that cuticle may be a target for plant defense. Differential regulation of genes associated with the antibacterial and immune response were also observed, suggesting that larvae of *M. saltuarius* may have evolved adaptations to cope with bacterial challenges in their host environments. The results from this study help to elucidate the underlying relationship between transcriptional plasticity and adaption mechanisms of herbivorous insects to host plants.

## Methods

### Larvae and host plant sample collection

Both male and female adults of *M. saltuarius* can spread exceeding 5 km throughout their whole life cycle [81]. To obtain representative *M. saltuarius* samples exclusively feeding on *P. koraiensis*, fourth-instar larvae of *M. saltuarius* were collected from Cangshi Forest Farm, located in Qingyuan Manchu Autonomous County, Liaoning Province (41°59′24.72′′N, 124°31′41.17′′E), in September 2019. To obtain representative *M. saltuarius* samples exclusively feeding on *P. tabuliformis*, fourth-instar larvae of *M. saltuarius* were collected from Cangshi Village, Fushun County, Liaoning Province (41°53′42.67′′N, 124°20′25.06′′E), on the same day in September, 2019. The two sample sites were approximately 40 km apart. Some of the larval samples were flash-frozen upon collection using liquid nitrogen, then transferred and stored at − 80 °C for subsequent RNA extraction. The remaining samples were transferred alive to the laboratory and reared on their corresponding hosts until development into instars. In addition, three sample logs of each host plant were collected for amino and fatty acid composition and content analysis.

### Host plant amino and fatty acid composition and content analysis

Three host plant samples from each *Pinus* spp. were used to investigate the composition and content of free amino acids according to the method described by Zeng et al. [82]. A 0.5 g sample powder was mixed with 50 mL hydrochloric acid (0.005 mol/L) for ultrasonic extraction for 35 min at 40 °C using ultrasonic cleaner. The homogenate was centrifuged at 15000 rpm for 15 min, then filtered with a 0.22 μm membrane. Amino acid content analysis was carried out by an A300 amino acid analyzer (membraPure Bodenheim, Germany) with a column packed with ion-exchange resin. Amino acid concentration was calculated by calibrating with external standards (Sinopharm Chemical Reagent, Beijing, China).

Similarly, three host plant samples from each *Pinus* spp. were used to investigate the composition and content of fatty acids. The crude fat were extracted from the samples with petroleum ether (boiling point 40–60 °C) using a Soxhlet extractor. Fatty acid methyl ester (FAME) was prepared according to the PORIM Test Method [83]. The GC-MS systems (Trace1310/ISQ, Thermo Fisher Scientific, USA) equipped with a flame ionization detector and a TG-5 MS capillary column (30 m × 0.25 mm × 0.25 μm) were used for fatty acid composition analysis. Chromatographic parameters were set as follows: the initial temperature was 80 °C for 1 min then raised to 200 °C at a rate of 10 °C/min, which was then increased to 250 °C at a rate of 5 °C/min, before being set to 270 °C at a rate of 2 °C/min and held for 3 min. The flow rate of carrier helium gas was 1.2 mL/min. FAMEs were identified by comparing their retention times with those of authentic standards (Sigma-Aldrich Chemie GmbH, Deisenhofen, Germany). Quantification of fatty acids was carried out based on the molecular weight of their corresponding FAMEs.

### Molecular identification of larvae

DNA barcode of mitochondrial *COI* was employed to ensure that the collected larvae belonged to the same species of *M. saltuarius*. A total of six *COI* sequences (677 bp) was amplified using the primer set LCO149/HCO2198 [84] from six representatives were obtained, i.e., three larvae feeding on each *Pinus* spp. The *COI* sequences of all individuals had little divergence, with the distances from 0.0 to 0.00897 falling within the range of genetic distance among *M. saltuarius* populations [85]. This, coupled with morphological characteristics, indicates that the larvae collected from different host plants all belong to *M. saltuarius*.

### Total RNA extraction and library construction

Total RNA was extracted with TRIzol reagent (Life Technologies, USA) according to the manufacturer’s instructions. RNA quality was assessed by a 1% agarose gel and the concentration was determined using a NanoDrop 2000 spectrophotometer (Thermo Fisher Scientific, USA). RNA integrity was examined using the Agilent Technologies 2100 Bioanalyzer system (Santa Clara, CA) with a RNA integrity number cutoff greater than 7.

To obtain the complete information of all transcripts, SMRT-seq (PacBio) was applied in this study. The best RNA sample (2 populations × 3 replicated samples) was selected and then pooled together in equal quantity for SMRT-seq. Full-length cDNA was synthesized using the SMRTer PCR cDNA Synthesis Kit (Biomarker, Beijing). PacBio Sequel II sequencing reactions from one SMRT cell (1–6 kb) were performed.

For Illumina RNA-seq, six RNA samples (2 populations × 3 replicated samples) were used. Then, the six libraries for sequencing were generated using NEBNext® Ultra™ RNA Library Prep Kit (NEB, Beverly, MA, USA) according to the manufacturer’s recommendations. Subsequently, the prepared libraries were sequenced on an Illumina NovaSeq 6000 platform (2 × 150 bp).

### PacBio sequencing data processing

SMRTlink 6.0 software was used to process the PacBio SMRT-seq raw reads. The CCS was obtained from the subreads.bam file. Sequencing adapters were trimmed, then clean CCS were classified into either full- or non-full-length isoforms based on cDNA primers and poly-A tail signal. To improve consensus accuracy, Iterative Clustering for Error Correction (ICE) and Arrow algorithm (https://downloads.pacbcloud.com/public/software/installers/smrtlink_5.0.1.9585.zip) were used to obtain high quality isoforms and full-length sequences. Additional nucleotide errors in consensus reads were corrected using the Illumina RNA-seq data with the software LoRDEC (Helsinki, Finland) [86]. BUSCO [87] was used to explore completeness according to conserved ortholog content.

### Structure analysis and annotation

Transcripts with lengths more than 200 nucleotides and with more than two exons were selected as lncRNA candidates. A combination of four pervasive coding potential assessment approaches was built, including CPC [88], CNCI [89], CPAT [90], and Pfam database [91], to identify lncRNA. The transcription factors were predicted using AnimalTFDB 2.0 [92]. Simple sequence repeats (SSRs) of the transcriptome were identified using MIcroSAtellite identification tool v1.0 (MISA) (http://pgrc.ipk-gatersleben.de/misa/).

Functional annotation of non-redundant transcripts was determined by searching in the public databases using BLASTX (v2.2.26) (cutoff E-value ≤1e-5) [93], including Nr, Nt (NCBI nucleotide sequences), KOG (euKaryotic Ortholog Groups), KEGG, Pfam, Swiss-Prot, and eggNOG (Non-supervised Orthologous Groups). Functional classification by GO analysis was conducted with the program Blast2GO (v2.5).

### Mapping and differential gene expression analysis

The pair-end Illumina reads were aligned to the reference transcriptome using Bowtie 2 (version 2.2.9) [94]. FPKM (fragments per kilobase of transcript per million fragments mapped) was used to estimate transcript expression levels in all samples [95]. Differential expression analysis between the larvae feeding on *P. koraiensis* and *P. tabuliformis* was implemented using the DEGseq R package (version 1.10.1) [96]. The threshold of DEGs was set at q-value < 0.05 and absolute fold change > 2. Heatmaps in this study were generated with a free online platform, OmicShare tools (http://www.omicshare.com/tools/) based on normalized FPKM data.

GO enrichment analysis of the DEGs was implemented using the GOseq R package (version 1.10.0) based on the Wallenius non-central hyper-geometric distribution to adjust gene length bias in DEGs [97]. The statistical enrichment of DEGs in KEGG pathways was tested using KOBAS (KEGG Orthology-Based Annotation System) (version v2.0.12) [98].

### Validation of DEGs with qRT-PCR

The real-time quantitative PCR (qRT-PCR) assay was conducted to validate the results of our transcriptome sequencing analysis. Reverse transcription was performed using the PrimeScript first strand cDNA synthesis kit (Takara, China) according to the manufacturer’s protocol. The gene-specific primers were designed using Primer5 (PREMIER Biosoft International, USA). The *ribosomal protein S3* gene was used for internal control. Quantitative reactions were performed on the Real-Time PCR Detection System (ABI 7500, Applied Biosystems, USA) with the SYBR Premix Ex Taq™ Kit (Takara, China). The qPCR setting was as follows: 95 °C for 5 min, followed by 40 cycles of 95 °C for 10 s and 60 °C for 30 s, melt curves stages at 95 °C for 15 s, 60 °C for 1 min, and 95 °C for 15 s. To check reproducibility, the qPCR reaction for each sample was performed in triplicate. Log_2_(fold change) qPCR was calculated by −∆∆Ct = − [(Ct_Pt_ – Ct_nom_) – (Ct_Pk_ – Ct_nom_)] [99].

## Supplementary Information

**Additional file 1: Figure S1.** Read length of CCS and FLNC. **a** CCS. **b** FLNC.

**Additional file 2: Figure S2.** The completeness of transcripts assessed by benchmarking universal single-copy ortholog (BUSCO). The x-axis represents the percentage of detected BUSCOs. The light blue diamond represents the complete (C) and single-copy (S) genes; the dark blue represents complete and duplicated (D) genes; the yellow diamond represents fragmented (F) genes; the red diamond represents the missing (M) genes. Total number of core genes queried was 1066.

**Additional file 3: Table S1.** Annotation of the 32,304 non-redundant transcripts.

**Additional file 4: Figure S3.** Species distribution of the top BLAST hits of the total homologous sequences.

**Additional file 5: Figure S4.** Gene ontology classification of non-redundant transcripts. The 13,144 transcripts were classified into three functional categories: molecular function, biological process and cellular component.

**Additional file 6: Figure S5.** KEGG pathway distributions of non-redundant transcripts. The genes according to KEGG metabolic pathway involved was divided into six branches: Metabolism, Genetic information processing, Environmental information processing, Cellular processes, Organismal systems, and Human disease.

**Additional file 7: Figure S6.** Number of transcript factors identified in the present study.

**Additional file 8: Table S2.** Summary of SSRs identified in the transcriptome of *Monochamus saltuarius.*

**Additional file 9: Table S3.** Annotation of the 2166 differentially expressed genes.

**Additional file 10: Figure S7.** Cluster analysis of differentially expressed genes. Different colors indicate different levels of gene expression. The firebrick color indicates upregulated expression, whereas the navy color indicates downregulated expression. Pk: the larvae feeding on *Pinus koraiensis*; Pt: the larvae feeding on *P. tabuliformis.*

**Additional file 11: Table S4.** List of KEGG pathways with *P*-value < 0.05.

**Additional file 12: Figure S8.** GO enrichment of differentially expressed genes. The 20 most enriched GO terms are shown together with their –log_10_(Q-value) and number of genes.

**Additional file 13: Table S5.** Primers used in qRT-PCR.

## Data Availability

Raw PacBio SMRT sequences and Illumina RNA-Seq data for this study have been deposited in the National Center for Biotechnology Information (NCBI) Sequence Read Archive (SRA) (http://www.ncbi.nlm.nih.gov/sra) (BioProject: PRJNA666339). All *COI* sequences obtained were submitted to NCBI GenBank (https://www.ncbi.nlm.nih.gov/genbank) (accession numbers: MW074321–MW074326).

## Abbreviations

ABC transporters: ATP-binding cassette transporters
AKR: Aldo-keto reductases
ALDH: Aldehyde dehydrogenase
AMPK: AMP-activated protein kinase
BUSCO: Benchmarking universal single-copy orthologs
CAT: Catalase
CCS: Circular consensus sequence
cDNA: Complementary DNA
CE: Carboxylesterases
CNCI: Coding-Non-Coding Index
COI: Cytochrome c oxidase subunit I
CPAT: Coding Potential Assessment Tool
CPC: Coding Potential Calculator
DEG: Differentially expressed gene
DNA: Deoxyribonucleic acid
ELOVL: Elongation of very long chain fatty acids protein
FAME: Fatty acid methyl ester
FAR: Fatty acyl-CoA reductase
FAS: Fatty acid synthase
FLNC read: Full-length non-chimera read
FPKM: Fragments per kilobase of transcript per million fragments mapped
GC content: Guanine-cytosine content
GC-MS: Gas Chromatography-Mass Spectromete
GILT: Gamma-interferon-inducible lysosomal thiol reductase
GO: Gene Ontology
GOBP: General odorant-binding protein
GPx: Glutathione peroxidase
Hsp: Heat shock proteins
ICE: Iterative Clustering for Error Correction
KEGG: Kyoto encyclopedia of genes and genomes
KOG: Eukaryotic orthologous groups
lncRNA: Long noncoding RNA
LPS: Lipopolysaccharide
LRR: Leucine-rich repeat
MISA: MIcroSAtellite identification tool
mRNA: Messenger RNA
MSRA: Peptide methionine sulfoxide reductase
Nr: NCBI non-redundant protein sequences
Nt: NCBI nucleotide sequences
OBP: Odorant-binding protein
P450: Cytochrome P450 monooxygenases
PCR: Polymerase chain reaction
Pfam: Protein family
PORIM: Palm Oil Research Institute of Malaysia
Prx: Peroxiredoxin
PWN: Pine wood nematode
RNA: Ribonucleic acid
RNA-Seq: RNA sequencing
ROS: Reactive oxygen species
qRT-PCR: Real-time quantitative PCR
SLC: Solute carriers
SMRT-seq: Single-molecule real-time sequencing
SNO: Senecionine N-oxygenase
SOD: Superoxide dismutase
SSR: Simple sequence repeats
STKs: Serine/threonine kinases
STPs: Serine/threonine phosphatases
TF: Transcription factor
TOR: Target of rapamycin
UGT: UDP-glycosyltransferases
UV: Ultraviolet
