# Supplementary figures and images for: Comparative transcriptome analysis of the newly discovered insect vector of the pine wood nematode in China, revealing putative genes related to host plant adaptation
Source: BMC Genomics. 2021 Mar 16;22:189. doi: 10.1186/s12864-021-07498-1 (PMC7968331; doi:10.1186/s12864-021-07498-1)

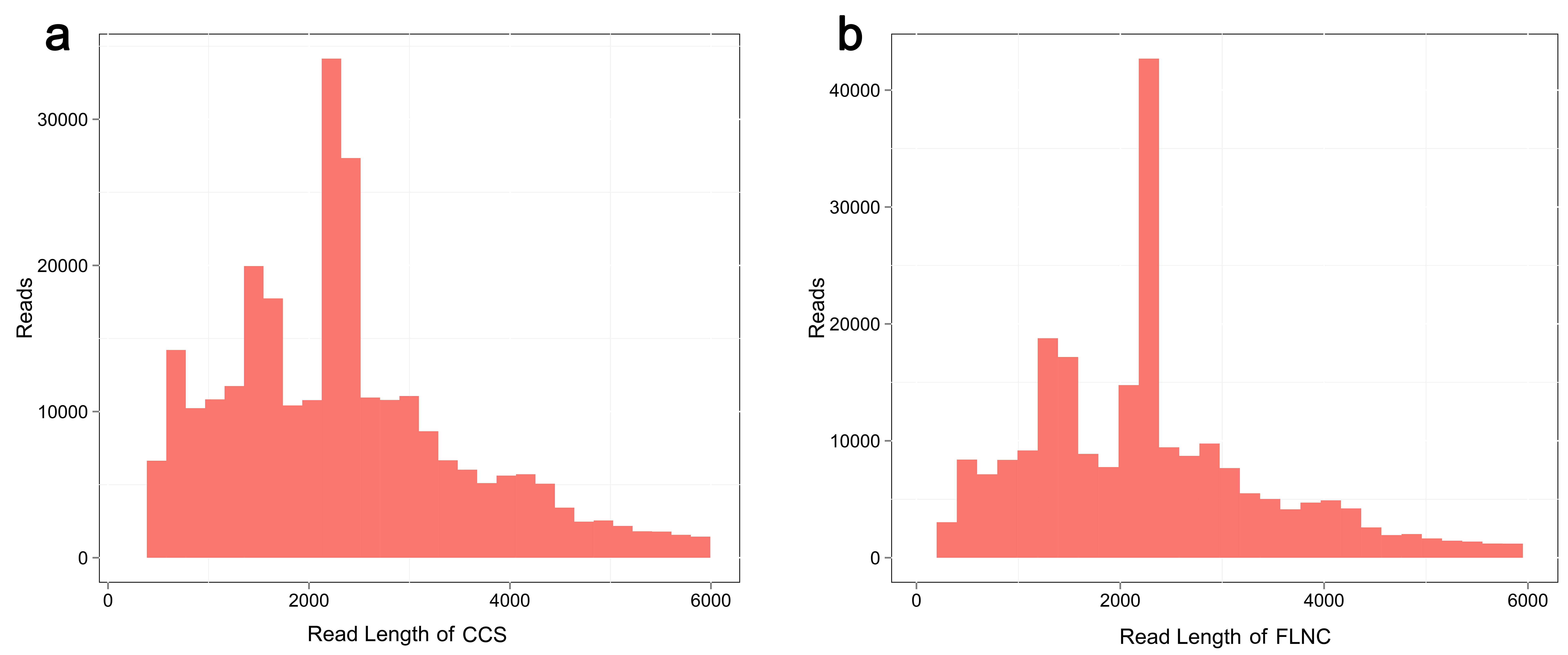

Supplement: Supplementary file 1 — Additional file 1: Figure S1. Read length of CCS and FLNC. a CCS. b FLNC. [file 12864_2021_7498_MOESM1_ESM.tif]

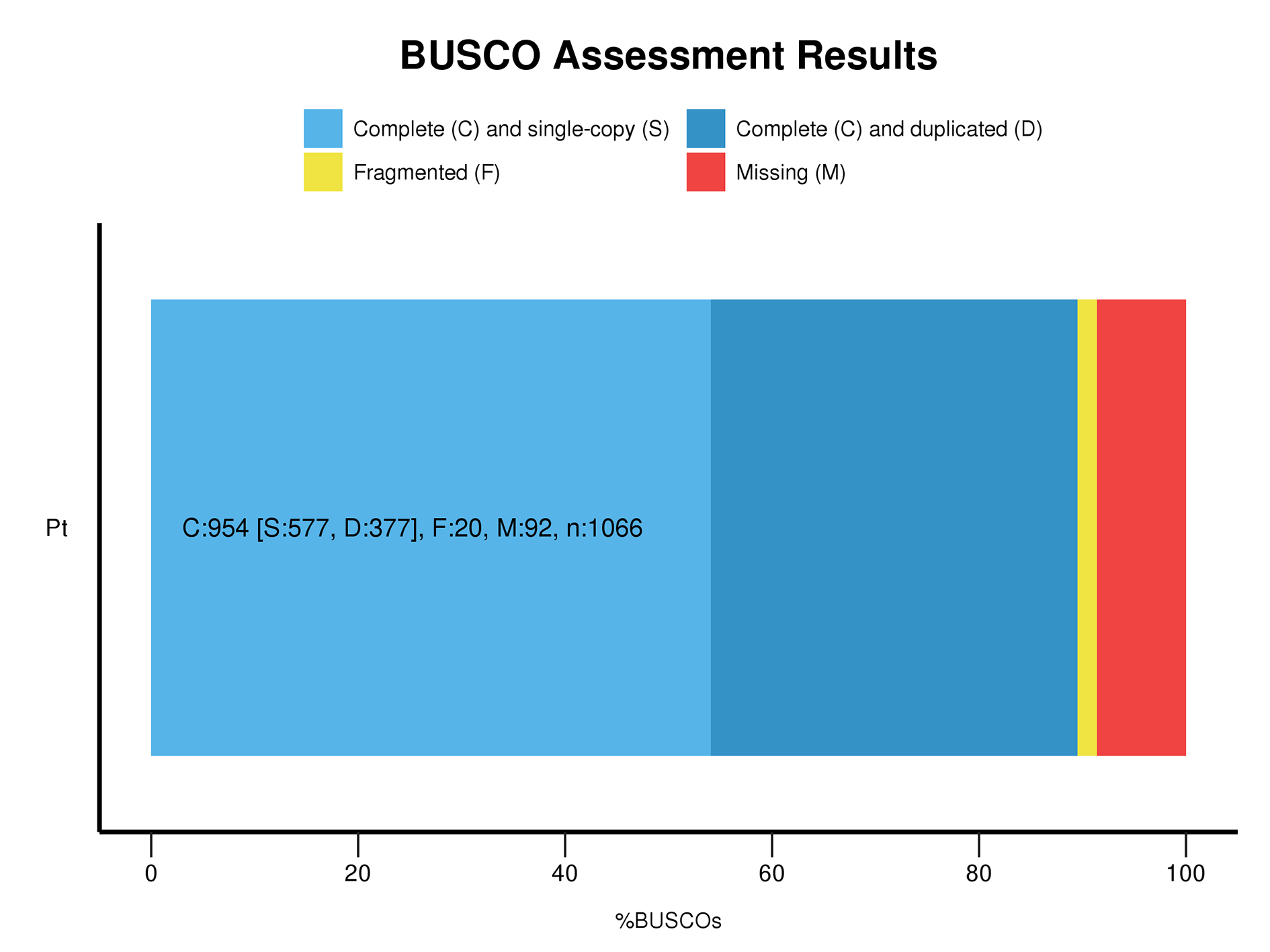

Supplement: Supplementary file 2 — Additional file 2: Figure S2. The completeness of transcripts assessed by benchmarking universal single-copy ortholog (BUSCO). The x-axis represents the percentage of detected BUSCOs. The light blue diamond represents the complete (C) and single-copy (S) genes; the dark blue represents complete and duplicated (D) genes; the yellow diamond represents fragmented (F) genes; the red diamond represents the missing (M) genes. Total number of core genes queried was 1066. [file 12864_2021_7498_MOESM2_ESM.tif]

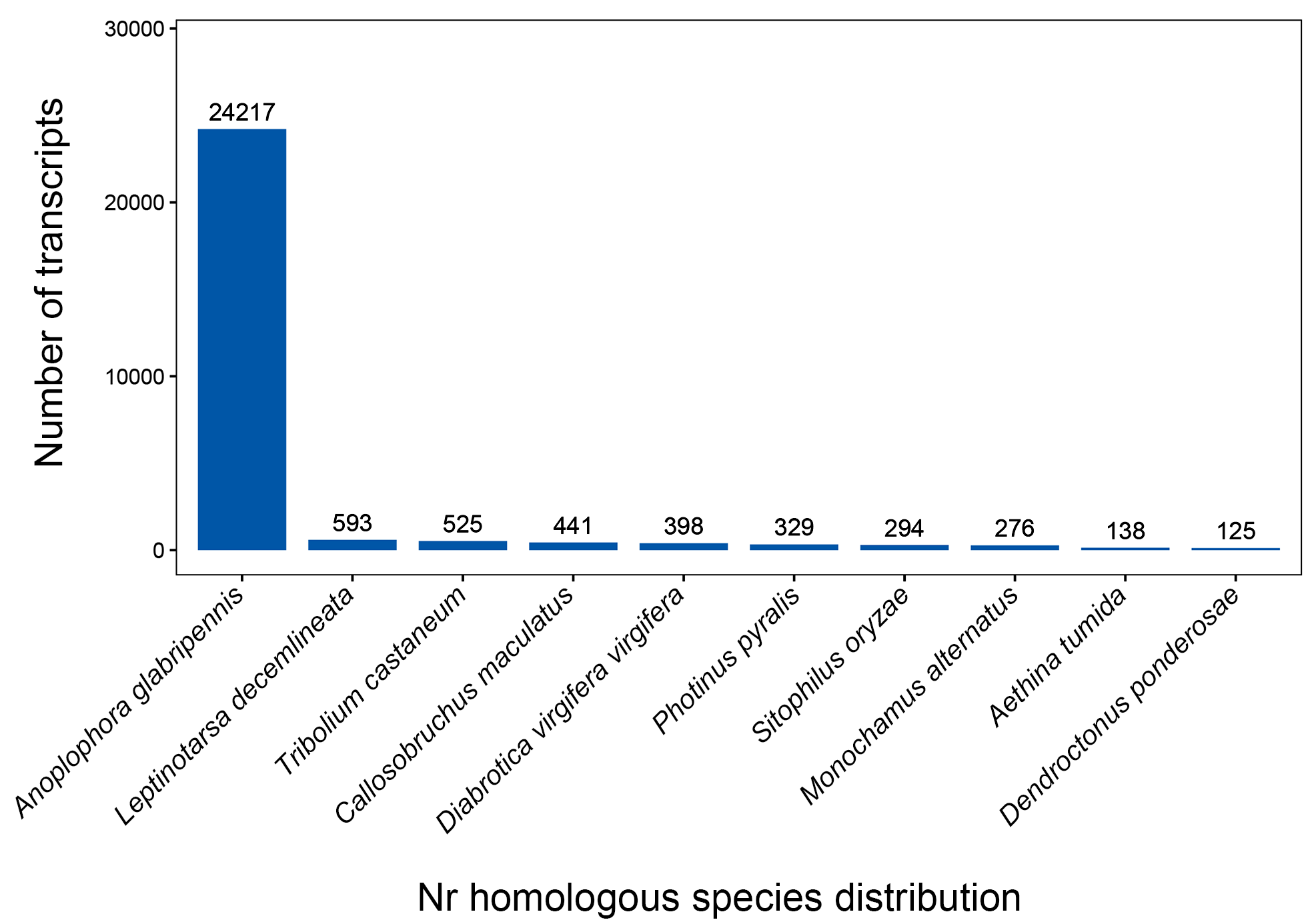

Supplement: Supplementary file 4 — Additional file 4: Figure S3. Species distribution of the top BLAST hits of the total homologous sequences. [file 12864_2021_7498_MOESM4_ESM.tif]

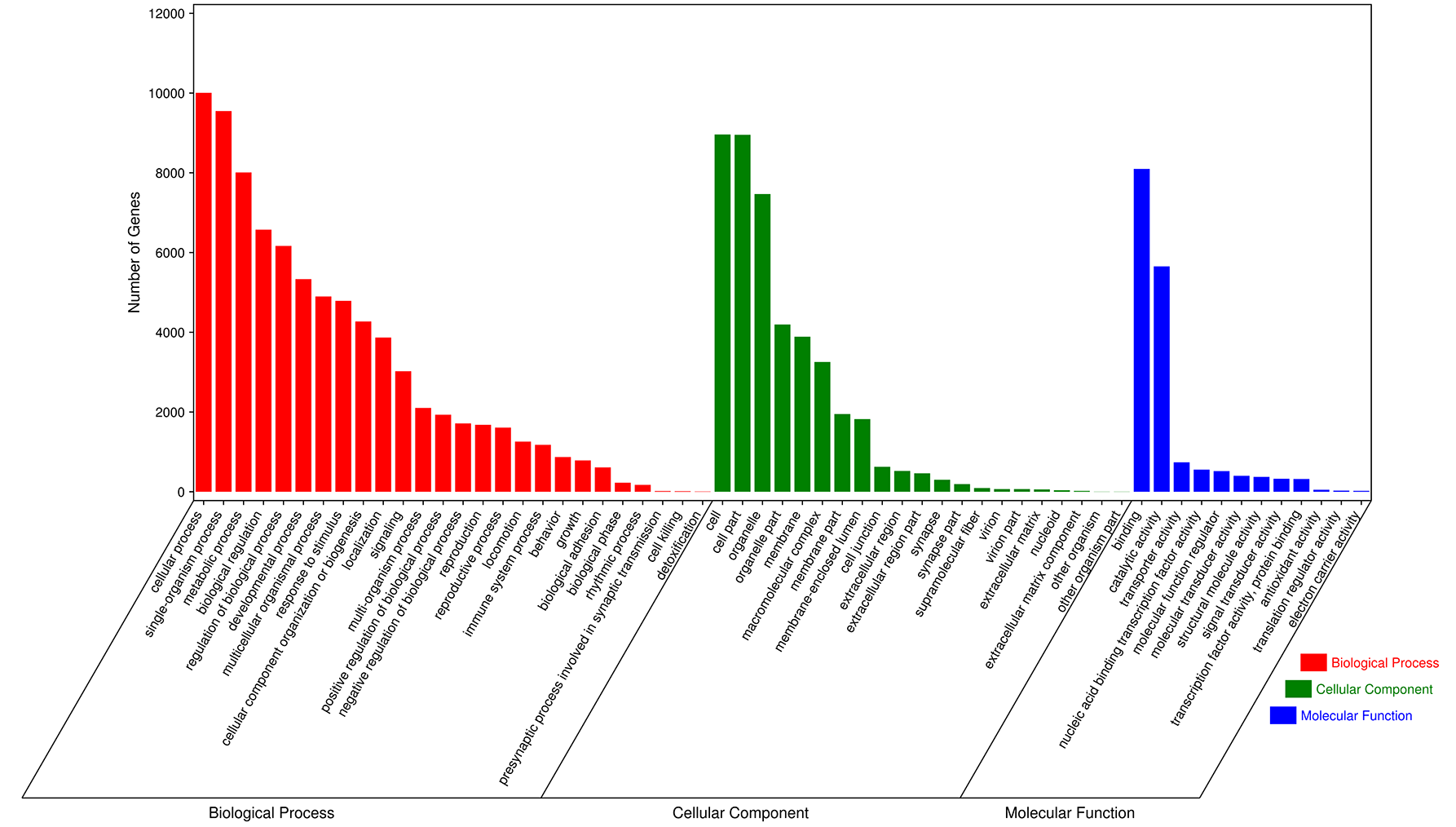

Supplement: Supplementary file 5 — Additional file 5: Figure S4. Gene ontology classification of non-redundant transcripts. The 13,144 transcripts were classified into three functional categories: molecular function, biological process and cellular component. [file 12864_2021_7498_MOESM5_ESM.tif]

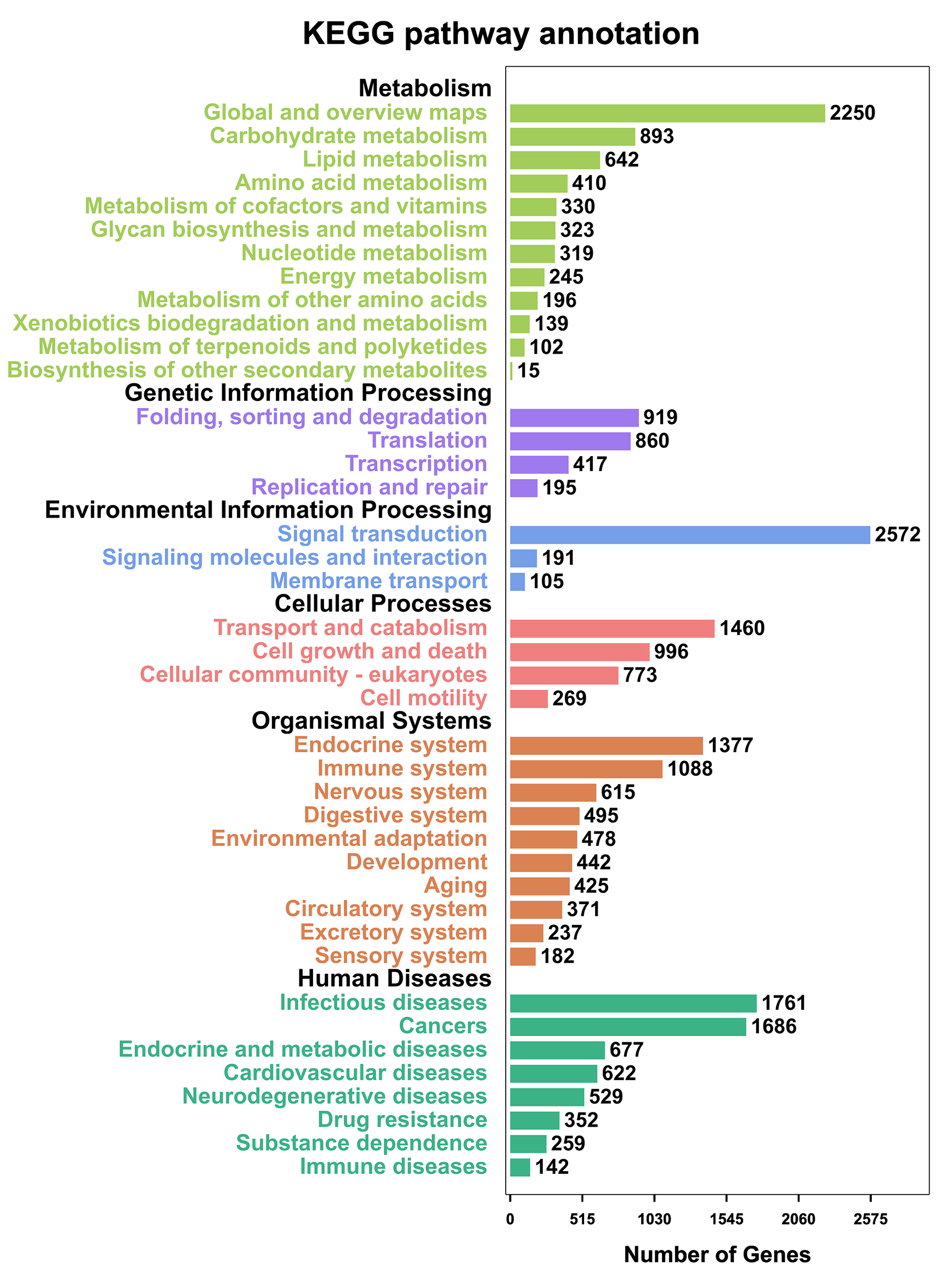

Supplement: Supplementary file 6 — Additional file 6: Figure S5. KEGG pathway distributions of non-redundant transcripts. The genes according to KEGG metabolic pathway involved was divided into six branches: Metabolism, Genetic information processing, Environmental information processing, Cellular processes, Organismal systems, and Human disease. [file 12864_2021_7498_MOESM6_ESM.tif]

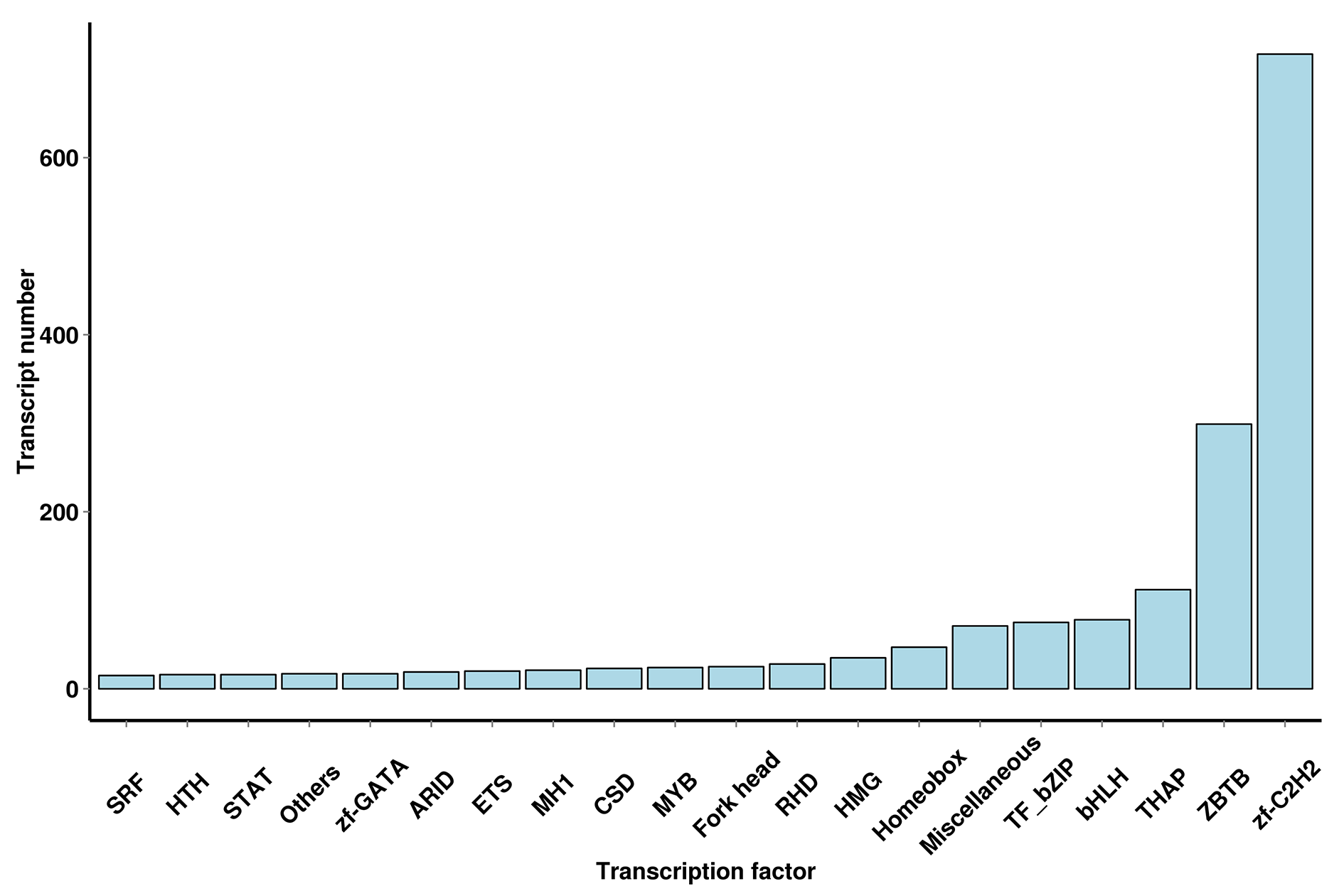

Supplement: Supplementary file 7 — Additional file 7: Figure S6. Number of transcript factors identified in the present study. [file 12864_2021_7498_MOESM7_ESM.tif]

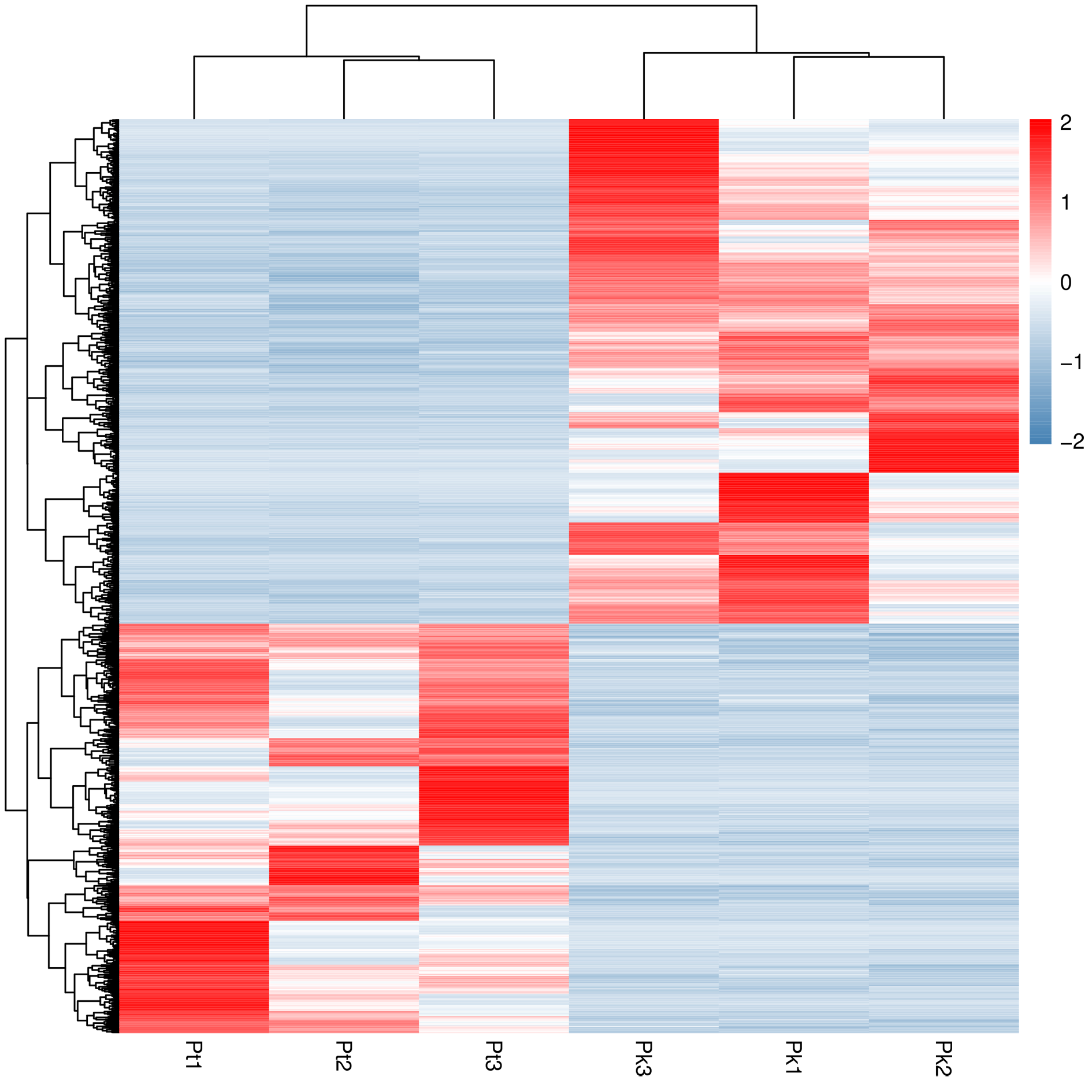

Supplement: Supplementary file 10 — Additional file 10: Figure S7. Cluster analysis of differentially expressed genes. Different colors indicate different levels of gene expression. The firebrick color indicates upregulated expression, whereas the navy color indicates downregulated expression. Pk: the larvae feeding on Pinus koraiensis; Pt: the larvae feeding on P. tabuliformis. [file 12864_2021_7498_MOESM10_ESM.tif]

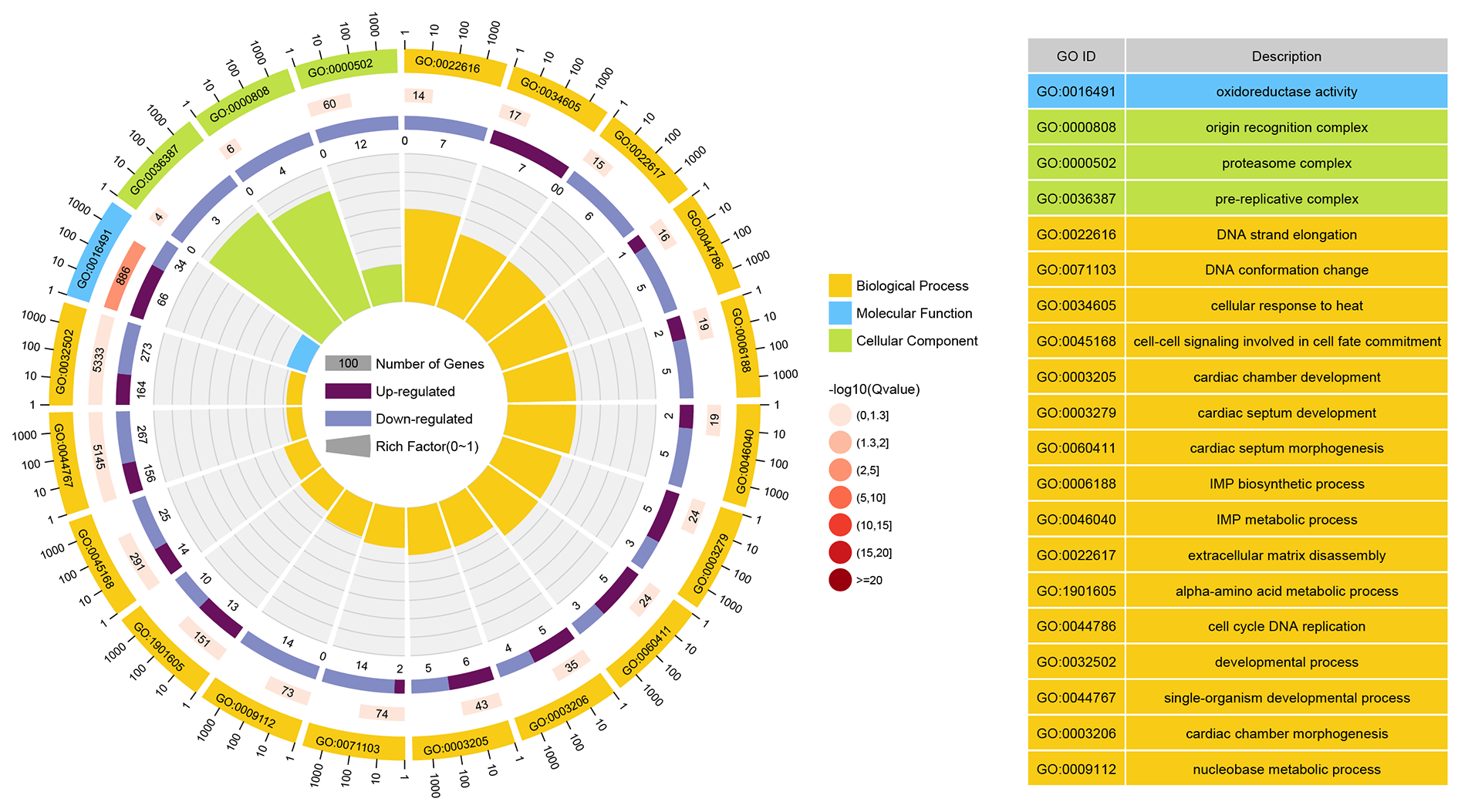

Supplement: Supplementary file 12 — Additional file 12: Figure S8. GO enrichment of differentially expressed genes. The 20 most enriched GO terms are shown together with their –log10(Q-value) and number of genes. [file 12864_2021_7498_MOESM12_ESM.tif]
