# Supplementary material for: Comparative transcriptome analysis of the newly discovered insect vector of the pine wood nematode in China, revealing putative genes related to host plant adaptation
Source: BMC Genomics. 2021 Mar 16;22:189. doi: 10.1186/s12864-021-07498-1 (PMC7968331; doi:10.1186/s12864-021-07498-1)
Supplement: Supplementary file 8 — Additional file 8: Table S2. Summary of SSRs identified in the transcriptome of Monochamus saltuarius. [file 12864_2021_7498_MOESM8_ESM.doc]

**Table S2.** **Summary of SSRs identified in the transcriptome of *Monochamus saltuarius***

| Searching item | Numbers |
| --- | --- |
| Total number of sequences examined | 31,530 |
| Total size of examined sequences (bp) | 106,022,512 |
| Total number of identified SSRs | 17,164 |
| Number of SSR containing sequences | 10,929 |
| Number of sequences containing more than 1 SSR | 3,799 |
| Number of SSRs present in compound formation | 1,398 |
| Mono nucleotide | 12,875 |
| Di nucleotide | 1,838 |
| Tri nucleotide | 2,231 |
| Tetra nucleotide | 187 |
| Penta nucleotide | 21 |
| Hexa nucleotide | 12 |
