# Supplementary material for: Comparative transcriptome analysis of the newly discovered insect vector of the pine wood nematode in China, revealing putative genes related to host plant adaptation
Source: BMC Genomics. 2021 Mar 16;22:189. doi: 10.1186/s12864-021-07498-1 (PMC7968331; doi:10.1186/s12864-021-07498-1)
Supplement: Supplementary file 11 — Additional file 11: Table S4. List of KEGG pathways with P-value < 0.05. [file 12864_2021_7498_MOESM11_ESM.doc]

**Table S4.** List of KEGG pathways with *P*-value < 0.05

| Pathway term | Pathway ID | Candidate genes with pathway annotation | All genes with pathway annotation | *P*-value |
| --- | --- | --- | --- | --- |
| DNA replication | ko03030 | 16 | 47 | 0 |
| Insect hormone biosynthesis | ko00981 | 12 | 60 | 0.001038 |
| Mismatch repair | ko03430 | 7 | 24 | 0.001171 |
| Tryptophan metabolism | ko00380 | 12 | 63 | 0.001622 |
| Cysteine and methionine metabolism | ko00270 | 13 | 76 | 0.002897 |
| Dilated cardiomyopathy (DCM) | ko05414 | 39 | 345 | 0.003422 |
| Hypertrophic cardiomyopathy (HCM) | ko05410 | 39 | 351 | 0.004602 |
| Longevity regulating pathway - multiple species | ko04213 | 23 | 179 | 0.004957 |
| Proteasome | ko03050 | 13 | 82 | 0.00568 |
| Legionellosis | ko05134 | 21 | 162 | 0.006394 |
| Prion diseases | ko05020 | 12 | 74 | 0.006439 |
| Cell cycle | ko04110 | 25 | 205 | 0.006856 |
| Cutin, suberine and wax biosynthesis | ko00073 | 7 | 32 | 0.006864 |
| Antigen processing and presentation | ko04612 | 13 | 86 | 0.008503 |
| Neuroactive ligand-receptor interaction | ko04080 | 8 | 43 | 0.010866 |
| Glyoxylate and dicarboxylate metabolism | ko00630 | 10 | 67 | 0.021395 |
| Glycine, serine and threonine metabolism | ko00260 | 8 | 51 | 0.02878 |
| Base excision repair | ko03410 | 7 | 43 | 0.033081 |
| Non-homologous end-joining | ko03450 | 4 | 19 | 0.043989 |
| Lipoic acid metabolism | ko00785 | 2 | 5 | 0.045113 |
| MAPK signaling pathway - fly | ko04013 | 35 | 363 | 0.048585 |
