# Supplementary material for: Comparative transcriptome analysis of the newly discovered insect vector of the pine wood nematode in China, revealing putative genes related to host plant adaptation
Source: BMC Genomics. 2021 Mar 16;22:189. doi: 10.1186/s12864-021-07498-1 (PMC7968331; doi:10.1186/s12864-021-07498-1)
Supplement: Supplementary file 13 — Additional file 13: Table S5. Primers used in qRT-PCR. [file 12864_2021_7498_MOESM13_ESM.doc]

**Table S5** Primers used in qRT-PCR

| Gene ID | Gene name | Forward primer | Reverse primer |
| --- | --- | --- | --- |
| Pt_transcript_10260 | UDP-glucuronosyltransferase 2B7-like | AGTGCTACCAACGGTTTCGT | ACTCGCCCAAAGTCTCAAGG |
| Pt_transcript_37639 | fatty acid synthase | TCGACGGGCGTGTTCTATTT | CCCGATACCGCTACAGAACC |
| Pt_transcript_907 | beta-1,4-mannosyltransferase egh | GGACGTCGGCTAAGTGACAA | TCGTAGCACCGATAACACCG |
| Pt_transcript_8747 | solute carrier family 25 member 38-A isoform X3 | ACAGGAAAGTGATGAGATGATGC | AGTTGACTTTCACGGTTTCAATTAC |
| Pt_transcript_45226 | cytochrome P450 315a1, mitochondrial | ACGCCCGGCTAAATAGAACC | GCCGCCACTAGCCTTGAATA |
| Pt_transcript_11651 | solute carrier family 25 member 35 | AATCCGGCTATCCTAACCTCA | ACTGGCTGCTAGACCTCCT |
| Pt_transcript_27055 | UDP-glucuronosyltransferase 1-9-like isoform X2 | ATGAAGTGCGTGCTCCTGTT | GCCAATTCTAGCCAAAGCGG |
| Pt_transcript_8102 | heat shock 70 kDa protein cognate 2 | TTCAAGCGGCCATTCTCAGT | GACAGCGCTTTGGTTGTCAG |
| Pt_transcript_29442 | esterase FE4 isoform X1 | AAGGACTGTTTAGAGCCGCC | GAGCCCTCGGTTATCGTGTT |
| Pt_transcript_4364 | cytochrome P450 6a8-like | ATTGCCCAGAGAGTGTGTGA | CGCAAACTCGTGGACCTTCT |
| Pt_transcript_7306 | heat shock protein 70 | ACTTTCGACTTGACGGGCAT | GTCTTTCTGCGACAATCGGC |
| Pt_transcript_6528 | heat shock protein 68-like | CCCGTATCTGCAGTCCCATC | TGATGTCCAAACCCTCCAGC |
| Pt_transcript_12744 | ribosomal protein S3 | TAGACACAGCCACCCGTCAT | CACTGCTTGGGAACAGTGGT |
